# Cost-effective options for increasing consumption of under-consumed food groups and nutrients in the USA

**DOI:** 10.1017/S1368980021000537

**Published:** 2022-03

**Authors:** Mary Brauchla, Victor L Fulgoni

**Affiliations:** 1 PepsiCo R&D Nutrition, Chicago, IL, USA; 2 Nutrition Impact LLC, 9275 D Drive North, Battle Creek, MI 49014, USA

**Keywords:** National Health and Nutrition Examination Survey, Cost, Vegetables, Fruits, Dairy, Whole grain, Vitamin C

## Abstract

**Objective:**

To identify the most cost-effective options/contributors of under-consumed food groups and nutrients in the USA.

**Design:**

Twenty-four-hour dietary recall data were used for the dietary sources of under-consumed food groups and nutrients. Costs were estimated using USDA National Food Price Database 2001–2004 after adjustments for inflation using Consumer Price Index.

**Setting:**

National Health and Nutrition Examination Survey, 2013–2016.

**Participants:**

A total of 10 112 adults aged 19+ years.

**Results:**

Top five cost-effective options for food groups were apple and citrus juice, bananas, apples, and melons for fruit; baked/boiled white potatoes, mixtures of mashed potatoes, lettuce, carrots and string beans for vegetables; oatmeal, popcorn, rice, yeast breads and pasta/noodles/cooked grains for whole grain; and reduced-fat, low-fat milk, flavoured milk and cheese for dairy. Top five cost-effective sources of under-consumed nutrients were rice, tortillas, pasta/noodles/cooked grains, rolls and buns, and peanut butter–jelly sandwiches for Mg; grits/cooked cereals, low- and high-sugar ready-to-eat (RTE) cereal, rolls and buns, and rice for Fe; low- and high-sugar RTE cereals, rice, protein and nutritional powders, and rolls and buns for Zn; carrots, margarine, other red and orange vegetables, liver and organ meats, butter and animal fats for vitamin A; and citrus juice, other fruit juice, vegetable juice, mustard and other condiments, and apple juice for vitamin C.

**Conclusions:**

Apple/citrus juice, white potatoes/carrots, oatmeal, RTE cereals and milk were the most cost-effective food sources of multiple under-consumed food groups and nutrients and can help promote healthy eating habits at minimal cost.

A healthy eating pattern primarily composed of fruits, vegetables, whole grains and low-fat dairy has been consistently recommended by the Dietary Guidelines for Americans (DGA) and other organisations^(1–3)^. Specific food group intake recommendations are provided in ChooseMyPlate^(4)^, and the USDA has also developed several food patterns that meet energy and food group recommendations for individuals with specific dietary needs or preferences (e.g. vegetarians)^(5)^. However, there is still a wide gap between food group recommendations and consumption: more than three-quarters of the US population currently do not meet daily intake recommendation for fruits (nearly 80 %), vegetables (nearly 90 %), whole grains (nearly 100 %) and dairy (nearly 80 %)^(1)^.

These gaps between recommended and actual food group consumption are significant because they translate to inadequate intakes of several essential nutrients. The 2015–2020 DGA^(1)^ identified several nutrients as ‘under-consumed’ including vitamin A, vitamin D, vitamin E, vitamin C, Ca, Fe (for certain age/gender groups), Mg, choline, K, and fibre; four of these nutrients (Ca, vitamin D, K and fibre) are classified as ‘nutrients of public health concern’ because intake at low levels is associated with adverse health outcomes. Many of these under-consumed nutrients are primarily found in fruits, vegetables, whole grains, and dairy, and previous public health messaging has promoted higher intakes of these food groups to close nutrient gaps.

Despite targeted public health messaging around these food groups intake remains well below the recommended levels^(1)^. The reasons underlying low food group intakes are multifactorial, but one contributor is the economic impact of choosing ‘healthier’ foods^(6–8)^. Food purchase decisions are often driven by food cost, which makes cost a major determinant of dietary intakes^(8–10)^. Rao *et al.* ^(11)^ conducted a meta-analysis and systematic review of twenty-seven studies and reported that healthier dietary patterns cost about $1·50 more per day (˜$550 more per year) compared to less healthy dietary patterns. In a recent analysis, we estimated that the USDA’s Healthy Food Patterns are generally more expensive than current US diet^(12)^. Additional studies have also come to the same conclusions: higher diet quality diets cost more and are associated with higher spending^(13–16)^. Therefore, there is a clear need for more solution-oriented research to identify low-cost healthier dietary options for low socio-economic status individuals and budget-conscious shoppers.

In a recently published analysis, we compared the cost of obtaining ‘shortfall nutrients’ including nutrients of public health concern (dietary fibre, vitamin D, Ca and K) from different food groups to identify cost-effective options for healthy and sustainable eating patterns for Americans^(17)^. The present analysis builds on our previous work by identifying the most cost-effective contributors to under-consumed food groups and additional nutrients in the USA.

## Methods

### Database and subjects

Data from What We Eat in America (WWEIA), the dietary component of the National Health and Nutrition Examination Survey (NHANES), were used^(18)^. NHANES is a continuous, nationally representative, cross-sectional survey of non-institutionalised, civilian US population conducted by the National Center for Health Statistics (NCHS) using a complex, multistage, probability sampling design, and the data are released in 2-year cycles. The present analysis combined two NHANES data cycles (NHANES 2013–2014 and NHANES 2015–2016). Day one 24-h dietary recall data from adults aged 19+ years (*n* 10 112) excluding pregnant and/or lactating females (*n* 218) and those with incomplete or unreliable dietary records as evaluated by the USDA Food Surveys Research Group staff (*n* 1461) were included in the analysis.

### Estimation of dietary intake

Dietary intake data were obtained via in-person 24-h dietary recall interviews administered using an Automated Multiple-Pass Method^(19)^. To determine food sources of food groups (fruits, vegetables, whole grains and dairy) and under-consumed nutrients (Mg, Fe, Zn, vitamin A and vitamin C), USDA’s WWEIA food groups were used at food main group (*n* 15), food subgroup (*n* 48) and food category (*n* 155) level^(20)^. Food category ‘Baby foods’ were excluded from the analysis, since the current analysis was focused on the adult population. Intake and composition of food groups were calculated using the NHANES cycle-specific Food Patterns Equivalents Databases (FPED)^(21)^. Nutrient intake and composition were determined using the respective Food & Nutrient Database for Dietary Studies (FNDDS) for each NHANES cycle^(22)^.

### Estimation of food cost

The USDA National Food Price Database (NFPD) was used to estimate cost of foods and nutrients from foods and beverages. The NFPD is derived from the Nielsen Homescan Consumer Panel and provides costs of all foods and beverages in NHANES 2001–2004^(23,24)^. Food cost for 2013–2016 was computed after adjusting the base food cost for inflation from 2001–2004 to 2013–2016 using Consumer Price Index (CPI) data from Bureau of Labor Statistics (BLS)^(25)^ as described previously^(12,17)^. The prices for foods and beverages were calculated per cup equivalent for dairy, fruits and vegetables, per ounce equivalent for whole grains, and per g, mg or µg for nutrients. NHANES food codes for 2013–2016 cycles were matched to NHANES 2001–2004 and then linked with NFPD to estimate base food cost. New food codes in NHANES 2013–2016 that were not present in NHANES 2001–2004 were hand-matched to the most closely matching food code. Cost-effectiveness was evaluated as amounts food groups and nutrients available per dollar.

### Statistics

All statistical analyses were performed with SAS software (version 9.4; SAS Institute Inc.). Data were adjusted for the complex sample design of NHANES using appropriate survey weights, strata, primary sampling units and day one dietary sample weights.

## Results

The top five cost-effective options for fruit were apple juice, citrus juice, bananas, apples and melons providing 2·73–4·20 cup equivalents per dollar. Except for apple juice, these were also the top contributors of daily intake of fruit. Together, the top five cost-effective options contributed 54·8 % (2·67 % + 13·6 % + 12·6 % + 17·8 % + 8·11 % contribution from apple juice, citrus juice, bananas, apples and melons, respectively) of daily intake of fruit (Table 1).

**Table 1:** Top ten cost-effective contributors of under-consumed food groups and their contribution to current intake*

Category no	Category	*n*	Rank by cost†	Cost-effectiveness (units of food group/dollar)‡		% Contribution to daily intake of food group		Rank by % daily intake of food group
				Mean	se	Mean	se	
Food group: total fruits (cup eq)								
7004	Apple juice	241	1	4·20	0·01	2·67	0·41	11
7002	Citrus juice	1069	2	4·19	0·07	13·6	0·6	2
6004	Bananas	1432	3	3·45	0·002	12·6	0·6	3
6002	Apples	1100	4	2·95	0·01	17·8	0·8	1
6014	Melons	489	5	2·73	0·07	8·11	0·87	4
7006	Other fruit juice	498	6	2·59	0·04	5·85	0·58	7
6016	Dried fruits	321	7	1·95	0·16	1·75	0·18	13
6008	Peaches and nectarines	284	8	1·86	0·08	2·44	0·36	12
6006	Grapes	480	9	1·85	0·001	3·55	0·29	10
7220	Smoothies and grain drinks	379	10	1·44	0·06	5·98	0·66	6
Food group: total vegetables excluding legumes (cup eq)								
6802	White potatoes, baked or boiled	476	1	3·71	0·08	4·06	0·36	8
6806	Mashed potatoes and white potato mixtures	793	2	3·16	0·07	5·81	0·46	4
6410	Lettuce and lettuce salads	2137	3	2·91	0·04	7·47	0·36	2
6404	Carrots	681	4	2·72	0·01	2·39	0·29	16
6412	String beans	450	5	2·45	0·04	2·52	0·25	15
6414	Onions	929	6	2·36	0·10	1·10	0·06	26
5002	Potato chips	1262	7	1·78	0·02	4·82	0·22	6
6804	French fries and other fried white potatoes	1586	8	1·78	0·04	6·73	0·25	3
6422	Vegetable mixed dishes	525	9	1·73	0·13	3·48	0·27	12
7008	Vegetable juice	119	10	1·72	0·20	0·90	0·13	29
Food group: whole grain (oz eq)								
4802	Oatmeal	575	1	5·13	0·10	8·50	0·52	4
5006	Popcorn	492	2	4·45	0·14	5·67	0·34	6
4002	Rice	1558	3	3·52	0·25	4·31	0·53	7
4202	Yeast breads	3636	4	3·27	0·12	32·9	1·0	1
4004	Pasta, noodles, cooked grains	296	5	3·06	0·63	2·18	0·45	10
4604	Ready-to-eat cereal, lower sugar (≤21·2 g/100 g)	806	6	2·40	0·13	9·67	0·55	2
3722	Peanut butter and jelly sandwiches (single code)	110	7	2·04	0·24	1·00	0·18	17
4602	Ready-to-eat cereal, higher sugar (>21·2 g/100 g)	962	8	2·03	0·06	8·93	0·52	3
5004	Tortilla, maize, other chips	1268	9	1·36	0·11	6·69	0·59	5
3720	Cheese sandwiches (single code)	72	10	1·30	0·37	0·55	0·10	20
Food group: total dairy (cup eq)								
1004	Milk, reduced fat	1467	1	4·50	0·02	11·0	0·7	2
1006	Milk, low fat	421	2	4·48	0·02	3·12	0·24	7
1206	Flavoured milk, low fat	32	3	2·81	0·12	0·48	0·14	34
1204	Flavoured milk, reduced fat	92	4	2·60	0·09	0·78	0·12	23
1602	Cheese	3150	5	2·35	0·03	21·2	0·7	1
1008	Milk, non-fat	436	6	2·34	0·01	4·17	0·40	6
1002	Milk, whole	1010	7	2·24	0·02	6·90	0·65	4
1202	Flavoured milk, whole	85	8	2·08	0·08	0·63	0·21	28
3720	Cheese sandwiches (single code)	72	9	1·99	0·03	0·49	0·07	33
1402	Milk shakes and other dairy drinks	127	10	1·71	0·09	1·21	0·20	20

*n*, number of consumers; NHANES, National Health and Nutrition Examination Survey.

* Data from NHANES 2013–2016 for adults aged 19+ years.

† Ranking of foods from most cost-effective to least cost-effective source.

‡ Cost-effectiveness was evaluated as amount of food group available per dollar; % contribution to daily intake of food group was calculated as intake of a food group from an individual food source/total daily intake of that food group from all dietary sources × 100.

Baked or boiled white potatoes, mixtures of mashed potatoes and white potato, lettuce, carrots, and string beans were top five cost-effective options for vegetables providing 2·45–3·71 cup equivalents per dollar. Together these contributed about 22·2 % of daily intake of vegetables (Table 1).

For whole grain, the top five cost-effective options were oatmeal, popcorn, rice, yeast breads and pasta/noodles/cooked grains providing 3·06–5·13 ounce equivalents per dollar. Yeast bread was the top contributor of whole grain and provided 32·9 % of daily intake. The top five cost-effective options of whole grain combined contributed to 53·6 % of total daily intake (Table 1).

Reduced-fat milk, low-fat milk, low-fat flavoured milk, reduced-fat flavoured milk and cheese were top five cost-effective options for dairy providing 2·35–4·50 cup equivalents per dollar. Cheese and reduced-fat milk were also the top two contributors of dairy and provided 21·2 % and 11·0 % of daily intake, respectively. The remaining three of the top five cost-effective options for dairy combined contributed to less than 5 % of total daily intake (Table 1).

Rice and rolls/buns were among the top five cost-effective sources for Mg, Fe and Zn, while high- and low-sugar ready-to-eat (RTE) cereals were also among the top five cost-effective sources of Fe and Zn and among the top ten for vitamin A. Oatmeal, the top cost-effective option for whole grains, was also among the top ten cost-effective contributors of Mg, Fe, Zn, and vitamin A. Other top five cost-effective sources of these minerals were tortillas, pasta/noodles/cooked grains and peanut butter–jelly sandwiches for Mg; grits for Fe; and protein/nutritional powders for Zn. The top five cost-effective sources of these minerals were relatively minor sources (except RTE cereals for Fe and Zn) and contributed to 3·79 % of daily Mg, 19·3 % of daily Fe, and 10·5 % of daily Zn. High- and low-sugar RTE cereals provided 14·8 % of daily Fe and 7·36 % of daily Zn (Table 2).

**Table 2:** Top ten cost-effective contributors of under-consumed nutrients and their contribution to current daily intake*

Categoryno	Category	*n*	Rank by cost†	Cost-effectiveness (units of nutrient/dollar)‡		% Contribution to daily intake of nutrients		Rank by % daily intake of nutrient
				Mean	se	Mean	se	
Nutrient: Mg (mg)								
4002	Rice	1558	1	301·0	4·0	1·09	0·09	28
4208	Tortillas	864	2	279·0	39·0	1·02	0·13	33
4004	Pasta, noodles, cooked grains	296	3	236·0	25·0	0·50	0·06	61
4204	Rolls and buns	1327	4	235·0	18·0	0·84	0·05	44
3722	Peanut butter and jelly sandwiches (single code)	110	5	234·0	4·0	0·34	0·04	79
4802	Oatmeal	575	6	221·0	6·0	1·08	0·07	29
2802	Beans, peas, legumes	1166	7	220·0	9·0	1·93	0·14	11
9802	Protein and nutritional powders	183	8	217·0	16·0	1·39	0·21	18
2804	Nuts and seeds	1744	9	181·0	4·0	6·31	0·38	1
5006	Popcorn	492	10	179·0	6·0	0·67	0·05	50
Nutrient: Fe (mg)								
4804	Grits and other cooked cereals	230	1	35·8	5·5	0·62	0·10	43
4604	Ready-to-eat cereal, lower sugar (≤21·2 g/100 g)	806	2	33·0	1·9	8·39	0·41	1
4204	Rolls and buns	1327	3	31·5	2·2	2·41	0·12	10
4602	Ready-to-eat cereal, higher sugar (>21·2 g/100 g)	962	4	23·0	0·8	6·39	0·26	2
4002	Rice	1558	5	19·6	0·6	1·51	0·13	18
4802	Oatmeal	575	6	13·7	0·4	1·43	0·10	19
4208	Tortillas	864	7	11·0	1·5	0·86	0·10	31
5204	Saltine crackers	339	8	10·1	0·4	0·23	0·02	83
4004	Pasta, noodles, cooked grains	296	9	9·85	0·52	0·44	0·04	55
2802	Beans, peas, legumes	1166	10	9·84	0·43	1·85	0·13	14
Nutrient: Zn (mg)								
4602	Ready-to-eat cereal, higher sugar (>21·2 g/100 g)	962	1	11·4	0·4	4·02	0·20	3
4604	Ready-to-eat cereal, lower sugar (≤21·2 g/100 g)	806	2	10·3	0·8	3·34	0·20	7
4002	Rice	1558	3	9·62	0·19	0·95	0·08	26
9802	Protein and nutritional powders	183	4	7·86	0·59	1·38	0·21	20
4204	Rolls and buns	1327	5	7·39	0·52	0·72	0·04	34
3702	Burgers (single code)	654	6	6·57	0·10	3·20	0·22	9
4802	Oatmeal	575	7	5·60	0·20	0·74	0·05	33
4004	Pasta, noodles, cooked grains	296	8	5·48	0·41	0·31	0·04	71
4208	Tortillas	864	9	5·47	0·73	0·55	0·07	53
1004	Milk, reduced fat	1467	10	5·24	0·03	1·79	0·12	16
Nutrient: vitamin A, RAE (mcg)								
6404	Carrots	681	1	2910·0	22	6·21	0·76	1
8004	Margarine	787	2	2309·0	53·0	1·33	0·12	26
6406	Other red and orange vegetables	314	3	1942·0	108·0	3·58	0·36	7
2010	Liver and organ meats	49	4	1682·0	679·0	0·79	0·31	37
8002	Butter and animal fats	986	5	807·0	10·0	1·37	0·09	23
4602	Ready-to-eat cereal, higher sugar (>21·2 g/100 g)	962	6	801·0	24·0	5·02	0·26	4
7008	Vegetable juice	119	7	734·0	89·0	0·93	0·20	35
1006	Milk, low fat	421	8	634·0	4·0	1·09	0·09	30
1004	Milk, reduced fat	1467	9	602·0	3·0	3·65	0·25	6
4802	Oatmeal	575	10	576·0	28·0	1·36	0·12	24
Nutrient: vitamin C (mg)								
7002	Citrus juice	1069	1	359·0	5·0	13·4	0·6	1
7006	Other fruit juice	498	2	186·0	5·0	4·86	0·45	6
7008	Vegetable juice	119	3	182·0	33·0	1·85	0·28	13
8406	Mustard and other condiments	1470	4	148·0	17·0	1·50	0·20	19
7004	Apple juice	241	5	123·0	6·0	0·90	0·15	31
7204	Fruit drinks	1267	6	116·0	7·0	6·68	0·38	3
6406	Other red and orange vegetables	314	7	102·0	15·0	1·51	0·31	18
6408	Dark green vegetables, excluding lettuce	970	8	78·0	2·7	5·62	0·48	4
7804	Enhanced or fortified water	65	9	72·8	3·5	0·82	0·14	32
9999	Not included in a food category	244	10	69·9	31·4	0·21	0·11	60

*n*, number of consumers; NHANES, National Health and Nutrition Examination Survey; RAE, retinol activity equivalents.

* Data from NHANES 2013–2016 for adults aged 19+ years.

† Ranking of foods from most cost-effective to least cost-effective source.

‡ Cost-effectiveness was evaluated as amount of a nutrient available per dollar; % contribution to daily intake of nutrient was calculated as intake of a nutrient from an individual food source/total daily intake of that nutrient from all dietary sources × 100.

Carrots, margarine, other red and orange vegetables, liver and organ meats, butter and animal fats were the top five cost-effective sources of vitamin A; and citrus juice, other fruit juice, vegetable juice, mustard and other condiments, and apple juice were the top five cost-effective sources for vitamin C. Carrots and citrus juice were also the top dietary contributors of vitamin A and vitamin C, providing 6·21 % and 13·4 % of daily intakes, respectively. The other top five cost-effective sources (rank 2–5) of vitamin A and vitamin C were minor contributors and together provided 7·07 % and 9·11 % of their respective daily intakes (Table 2).

Oatmeal, the top cost-effective option for whole grains was also among the top ten cost-effective contributors of Mg, Fe, Zn and vitamin A. Similarly, RTE cereals were also among the top ten cost-effective contributors of Fe, Zn and vitamin A, in addition to being among top five cost-effective options for whole grain. Milk, in addition to being the top cost-effective option for dairy, was among the top ten cost-effective contributors of Zn and vitamin A while carrots, the fourth most cost-effective option for vegetables, was the top cost-effective contributor of vitamin A. Citrus juice was the second most cost-effective option for fruit and the most cost-effective source of vitamin C (Tables 1 and 2).

## Discussion

In the present analysis of NHANES 2013–2016 using a recent nationally representative sample of US adults, the most cost-effective sources of fruits, vegetables, whole grains and dairy were apple juice, white potatoes, oatmeal and reduced-fat milk, respectively. To the best of our knowledge, this is the first analysis of nationally representative, non-institutionalised population of US adults identifying the cost-effective contributors to key food groups. Additionally, in the present analysis, rice, grits and cooked cereals, RTE cereals, carrots, and citrus juice were the most cost-effective sources of Mg, Fe, Zn, vitamin A and vitamin C, respectively.

Numerous studies comparing the cost of foods and diets have indicated that healthier options cost more than less healthy options^(11–16)^. Cost of food is a major factor in food choice and has therefore been a significant barrier to healthy eating, especially among low socio-economic groups^(26–28)^. However, few studies have compared the cost of different foods or identified lower cost options for under-consumed food groups or nutrients.

Previous research identified potatoes and beans as the lowest-cost sources of K and fibre among frequently consumed vegetables^(29)^. In addition, our results suggest that potatoes are the most cost-effective source of vegetables and more than twofold less expensive than the most consumed vegetable option ‘other vegetables and combinations’ (3·71 *v*. 1·68 cups equivalents per dollar). For whole grains and dairy, the most cost-effective contributors were oatmeal and reduced-fat milk which were 36 % and 48 % less expensive, respectively, than the most common dietary sources of whole grains and dairy, namely bread and cheese. In the present analysis, the most cost-effective food sources of under-consumed minerals Mg, Fe and Zn were also less expensive than their respective most common dietary sources. In a dietary modelling study aimed to eliminate shortfall of fruit intake among children of 4–18 years old, a combination of 100 % fruit juice + whole fruit was found to be 4·3 % less expensive option than whole fruit alone^(30)^. In the present study, we found that apple juice was the most cost-effective source of fruit intake and was about 30 % less expensive (42 % more fruit cup eq per dollar) than apples, the top dietary source of fruit.

Foods such as oatmeal, RTE cereals, milk, carrots and citrus juice were among the most cost-effective food sources of multiple under-consumed food groups and nutrients. The present analysis shows that per dollar, oatmeal provides 5 oz eq (>100 % of daily recommended amount) of whole grain, 221 mg (>50 % RDA) of Mg, 14 mg (>75 % RDA) of Fe, 5·6 mg (>50 % RDA) of Zn and 556 µg retinol activity equivalents (RAE) (>60 % RDA) of Zn along with other nutrients. Similarly, for each dollar spent, RTE cereals provide >2 oz eq whole grain (>50 % of daily recommended amount), 33 mg (>100 % RDA) of Fe, >10 mg (>90 % RDA) of Zn and 800 µg RAE (>85 % RDA) of vitamin A; and milk provided 4·5 cups eq (>100 % of daily recommended amount) of dairy, >5 mg (>40 % RDA) of Zn and >600 µg RAE (>60 % RDA) of vitamin A. Additionally, these foods are meaningful sources of other key nutrients not analysed in this report. These foods could easily be included (if not already in) in the USDA’s Thrifty Food Plan (TFP) which is the national standard for minimal cost diet to help meet dietary recommendations and serves as the basis for the allotment of Supplemental Nutrition Assistance Program (SNAP) benefits^(30)^. The average American family spends about 25 % more on foods than in the TFP and even low-income families spend more than the TFP, highlighting the importance of communicating low-cost food and beverage options to both low SES and budget-conscious shoppers.

This study has several strengths and limitations. Using a large, nationally representative database to estimate nutrient cost is a major strength of the present study. However, using self-reported 24-h dietary recall data may be a limitation, as it is subject to over- or under-reporting and may not provide a true picture of usual eating habits. The USDA NFPD assumes that all foods were purchased at retail and prepared at home, restaurant prices were not included, and may not reflect seasonal, geographic/location-related differences in food price/diet cost. Some food codes in NHANES 2013–2016 had to be hand-matched to the closest available food codes in NHANES 2001–2004 database, and this process may have introduced inaccuracies in cost estimations. Additionally, there may be inaccuracies in the adjustment for inflation, based on CPI/BLS food categories as price differentials within a category would not have been captured.

In conclusion, apple/citrus juice, white potatoes/carrots, oatmeal, RTE cereals and milk were the most cost-effective food sources of multiple under-consumed food groups and nutrients. This information should be included in dietary recommendations to ensure healthy eating habits at minimal cost to the consumer.
